# Mind the gap: a German case study on the discrepancy between geographic accessibility and real-world utilization of botulinum toxin therapy

**DOI:** 10.3389/fneur.2026.1715279

**Published:** 2026-02-18

**Authors:** Tristan Koelsche, Robin Jansen, Lars Masanneck, Marc Pawlitzki, Sven G. Meuth, John-Ih Lee, Philipp Albrecht

**Affiliations:** 1 Department of Neurology, Medical Faculty and University Hospital Düsseldorf, Heinrich Heine University Düsseldorf, Düsseldorf, Germany; 2 Department of Neurology and Neurophysiology, GFO Clinics Rhein-Berg, Bergisch Gladbach, Germany; 3 Department of Neurology, Kliniken Maria Hilf, Mönchengladbach, Germany

**Keywords:** botulinum toxin, health services research, healthcare accessibility, spasticity, treatment gap

## Abstract

**Background:**

Botulinum toxin A (BoNT-A) is widely used in the treatment of neurological disorders. Despite guideline recommendations, particularly regarding BoNT-A injections for post-stroke spasticity, current evidence shows that many patients in Germany do not receive BoNT-A therapy.

**Methods:**

An analysis of travel time to neurological Botulinum Toxin (BoNT-A) centers was conducted based on German hospital quality reports. Additionally, the German Botulinum Toxin Working Group (Arbeitskreis Botulinumtoxin e. V.; AK-BoNT) performed an anonymous online survey of its members, who treat with BoNT, between November 2023 and March 2024 to document experiences, identify perceived barriers, and explore potential improvements in BoNT-A therapy.

**Results:**

The geospatial analysis showed theoretical accessibility to specialized neurological BoNT−A centers in Germany with 96.72% of the population residing within a 60-min drive of a qualified facility. Of 632 invited AK-BoNT members, 191 completed the survey in full (30.2% response rate). 79.6% of the respondents worked in neurology and two thirds of the respondents had more than 10 years of experience with BoNT-A. Although more than 60% of respondents rated BoNT-A therapy as effective regardless of indication, many pointed to structural and economic barriers, including inadequate reimbursement pathways and limited multidisciplinary collaboration.

**Conclusion:**

The results indicate that BoNT-A is recognized and provides therapeutic benefit from the perspective of the treating physician, but structural and economic barriers hinder the use of this therapy. Interestingly, even in a well-resourced system with broad geographic access to specialized care, significant treatment gaps may persist. Recommendations include standardized reimbursement structures, improved education, and increased cross-sector collaboration.

## Introduction

The use of botulinum toxin A (BoNT-A) is an established and highly effective therapeutic procedure for various neurological conditions, including spasticity, dystonias, and chronic migraine (CM) (1). International guidelines recommend its use, particularly early and adequately dosed injections to reduce complications like contractures, pain, and functional limitations in conditions such as post-stroke spasticity (2).

Despite its proven efficacy, a significant “treatment gap” often exists between guideline recommendations and clinical reality. Many patients who could benefit from BoNT-A therapy do not receive it in a timely or adequate manner. This issue is not unique to any single country but represents a global challenge in healthcare delivery.

This study uses Germany as a case study to investigate the factors contributing to this treatment gap. Germany provides a valuable model, as it has a well-resourced healthcare system and established clinical guidelines, yet evidence points to a significant undersupply of BoNT-A therapy (3). The German system is characterized by complex, regionally fragmented reimbursement structures and a division between outpatient and inpatient care, which can create barriers to access. By examining this system, we aimed to identify specific structural, economic, and educational hurdles that may be relevant to other healthcare systems worldwide.

Therefore, this study, conducted by the German Botulinum Toxin Working Group (Arbeitskreis Botulinumtoxin e. V., AK-BoNT), aimed to identify factors influencing the use of BoNT-A in Germany. We combined a geospatial accessibility analysis with a national survey of expert clinicians to create a comprehensive picture of the care landscape. The goal is to provide actionable insights for clinicians, hospital administrators, and health policymakers internationally on how to optimize patient access to this important therapy.

## Methods

### Ethics statement

The study was approved by the ethics committee of the Heinrich-Heine-University Düsseldorf (Study number: 2025-3254).

### Accessibility analysis of neurological botulinum toxin centers

An analysis of the accessibility (driving minutes by car with average traffic volume) of centers that offer neurological BoNT-A therapy was conducted. This was based on data from the year 2022 reporting of the mandatory hospital quality reports, as required by federal law (§ 136b Abs. 1 S. 1 Nr. 3 SGB V). (The hospital quality reports are only used partially or in excerpts here. A complete, unchanged presentation of the hospital quality reports can be obtained at www.g-ba.de/qualitaetsberichte, prepared in the German Hospital Directory^1^ by “Deutsche Krankenhaus TrustCenter und Informationsverarbeitung GmbH”). This second methodological step served to geographically map the regional care situation and to contextualize it with the survey results.

### Data basis, geocoding, and calculation of isochrones

First, all hospitals listed in the German Hospital Directory with a neurology department and at least 4 cases with the Operation and Procedure Classification System (OPS) code “6-003.8: Application of medication, List 3: Botulinum toxin” in the 2022 reporting year were identified as facilities with proven neurological BoNT-A therapy. Their address details (e.g., street, postal code) were converted into geographical coordinates (latitude and longitude) (“geocoding”) and standardized for further analysis steps. The analyses were performed using Python 3.8.8 (Python Software Foundation, Delaware, United States) with the pandas package version 1.3.4. All services used are based on data from the OpenStreetMap (OSM) project, which forms the basis for all displayed maps. The spatial analysis and manipulation of geometric objects were carried out with the Rasterio package version 1.1.120 and the Shapely package version 1.7.1. The geocoding of addresses was done with the Geopy package version 2.2.022 using the Nominatim geocoder for OSM data.

Following established procedures for care analysis, travel time polygons (isochrones) were then created for each facility, analogous to previous work (4, 5). For this, route-based geoinformation systems (local installation of openrouteservice) were used, which determine how far one can travel by car within a certain driving time (30, 60, 90 min) based on the road network, speed limits, and average traffic volume. Each time interval was mapped in a separate polygon.

### Population coverage

To quantify the proportions of the population that can reach a center within less than 30, 60, or 90 min, respectively, estimated population data for the year 2025 [publicly available Global Human Settlement Layer population grid (GHS-POP R2023A)] (6) were overlaid with the isochrones. This allowed for the determination of the absolute and relative population size located within a specific travel time interval. Subsequently, an extrapolation to the total population of Germany was performed. The results were visualized using matplotlib 3.6.3. The hospital quality reports are used here in conjunction with other sources of information. The recommendations and results presented therefore do not represent an authentic reproduction of the quality reports. A complete presentation of the hospital quality reports can be obtained at www.g-ba.de/qualitaetsberichte.

### Sample and recruitment

In addition to the accessibility analysis, an online survey was conducted among the members of the AK-BoNT. The questionnaire was created using SoSci Survey (7) and made available to the participants at www.soscisurvey.de. The invitation to the study was sent via email, which contained a link to the online questionnaire. Participation was voluntary and anonymous; written consent for the evaluation of the data was obtained via electronic agreement at the start of the survey. The study protocol stipulated the inclusion of only physicians who use BoNT-A in their practice or clinical facility or who have a fundamental interest in this form of therapy. The target population consisted of office-based and hospital-based members of the AK-BoNT from various specialties [e.g., neurology, ear nose and throat (ENT), urology, ophthalmology, general practitioner (GP)]. The survey was conducted over a period from November 2023 to March 2024.

### Instrument and questionnaire

The online questionnaire included both closed and semi-open questions. Information was collected on sociodemographic data, details of professional experience and patient numbers, as well as assessments of the current care situation. In addition, the questionnaire contained items on specific aspects of BoNT-A therapy, such as preferred preparations, common side effects, perception of effectiveness in various indications, and perceived hurdles in billing. Participants could also enter suggestions for training formats, improvements to the reimbursement structure, and other optimization possibilities in free-text fields.

### Data preparation and analysis

After the survey was completed, the data was anonymized, exported, and checked for plausibility. Questionnaires with significant missing or duplicate entries were excluded. The statistical analysis was descriptive: frequencies, means, standard deviations, medians, and interquartile ranges were calculated and presented in tabular form. The data analysis and the creation of tables and graphics were carried out with SPSS Version 28.0.0.0 (IBM). All collected data were treated confidentially and used exclusively for scientific purposes. It was not possible to trace individual participants based on the survey results.

## Results

### Accessibility of neurological botulinum toxin centers

**Figure 1:**
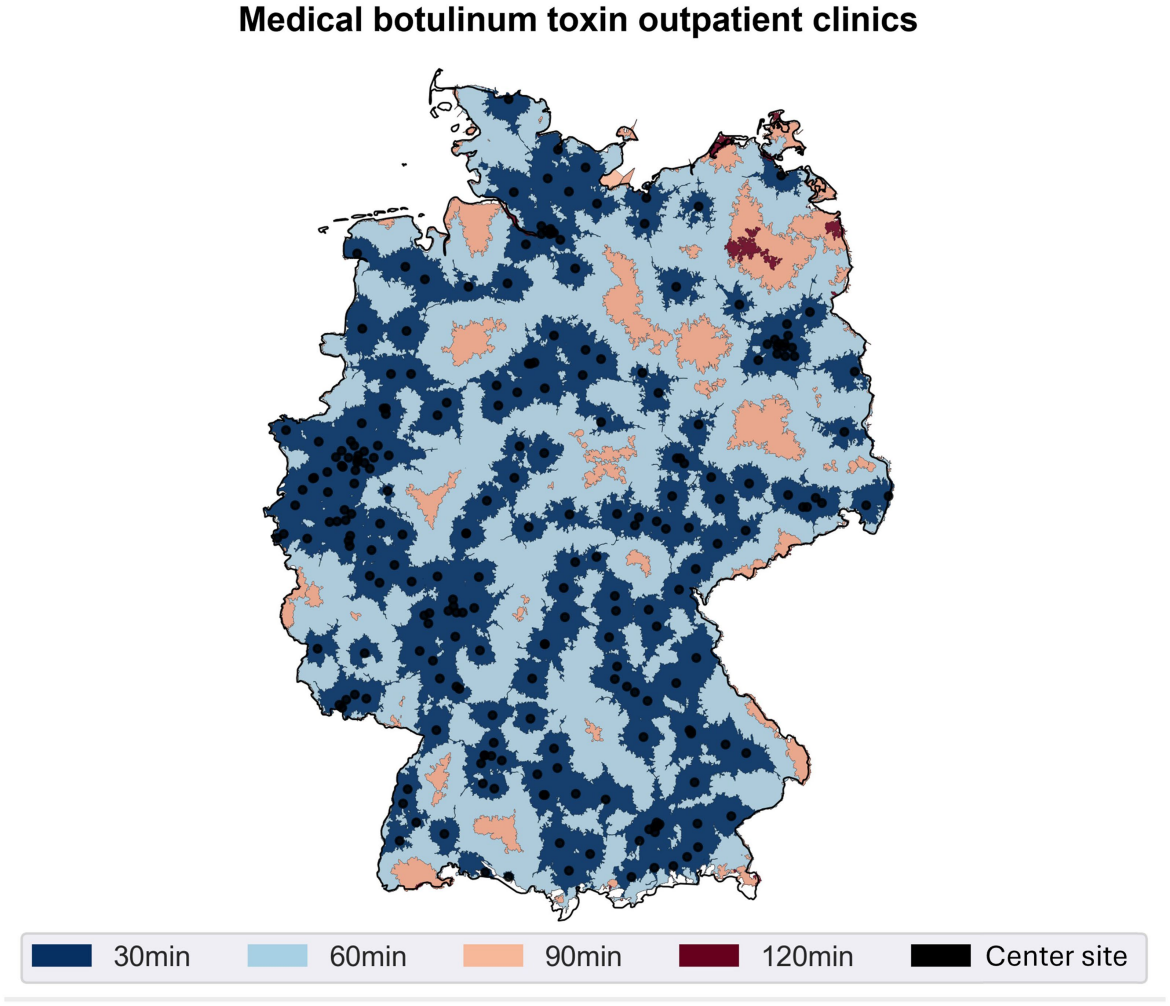
Accessibility of neurological botulinum toxin centers. Distribution of special outpatient clinics offering neurological botulinum toxin therapy according to the German Hospital Directory 2022 (black dots), as well as the colored areas indicating different maximum travel times by car (30, 60, 90, and 120 min). By overlaying these isochrones, it becomes clear that the majority of the population is within a 90-min drive of at least one treatment center.

A geospatial analysis was conducted to determine the accessibility of specialized neurological Botulinum Toxin (BoNT-A) centers across Germany (Figure 1). Based on mandatory hospital quality reports from 2022, centers were identified as facilities with a neurology department reporting a minimum of four cases using the specific procedural code for BoNT-A application. Figure 1 visually represents the results of this analysis, mapping the travel time required to reach the nearest treatment center by car under average traffic conditions. The analysis reveals extensive geographic coverage throughout the country. A substantial majority of the German population, 68.87% (approximately 55.68 million people), resides within a 30-min drive of a specialized center. Expanding the travel time to 60 min increases this coverage significantly, encompassing 96.72% of the population. At a maximum travel time of 90 min, the analysis demonstrates that 99.81% of the population can access a neurological BoNT-A center, indicating near-comprehensive geographic availability of these specialized services.

### Questionnaire

In total, the online questionnaire was sent to 632 members of the AK-BoNT, of whom 191 fully completed it. This corresponds to a response rate of 30.2%.

### Demographic data

**Figure 2:**
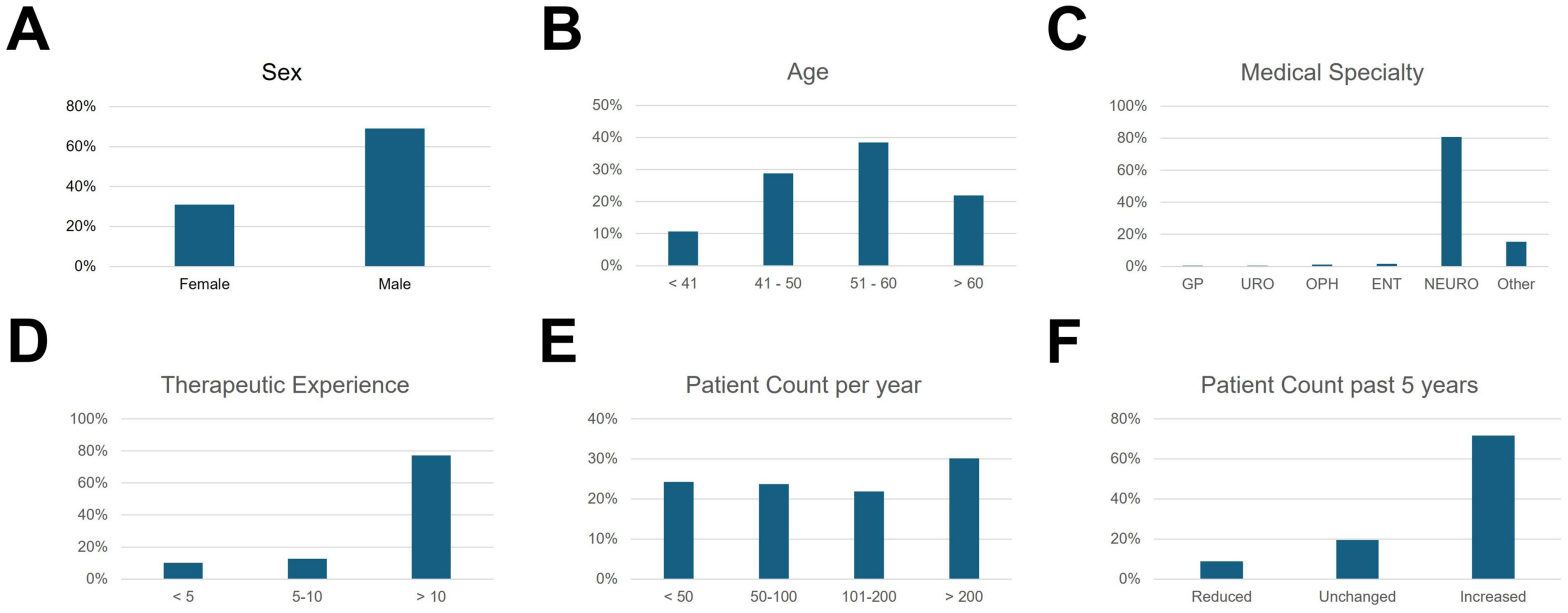
Demographic and professional characteristics of the respondents. The bar charts show the percentage of respondents (*n* = 191). **(A)** Gender of respondents, **(B)** age of respondents, **(C)** medical specialty, **(D)** duration of treatment experience with botulinum toxin in years, **(E)** annual number of patients treated, and **(F)** change in the number of patients within the last 5 years.

Of the practitioners, 29.8% were female and 70.2% were male. The age distribution was as follows (*n* = 188): 10.5% were up to 40 years old, 28.3% were between 41 and 50 years, 37.7% were between 51 and 60 years, and 21.5% were over 60 years old (Figure 2). The majority (79.6%) of respondents practice neurology. Other specialties included ENT (1.6%), urology (0.5%), ophthalmology (1.0%), general medicine (0.5%), and others (11.0%). 67% of respondents stated they had at least 10 years of experience with BoNT-A injections; only 1% do not perform the injections themselves. Per year, 21.5% of respondents treated fewer than 50 patients, 20.9% between 50 and 100, 19.4% between 101 and 200, and 26.7% more than 200. For 71.6%, the number of treated patients increased during the past 5 years, 8.9% recorded a decrease, and 19.5% reported unchanged numbers.

### Reimbursement aspects

**Figure 3:**
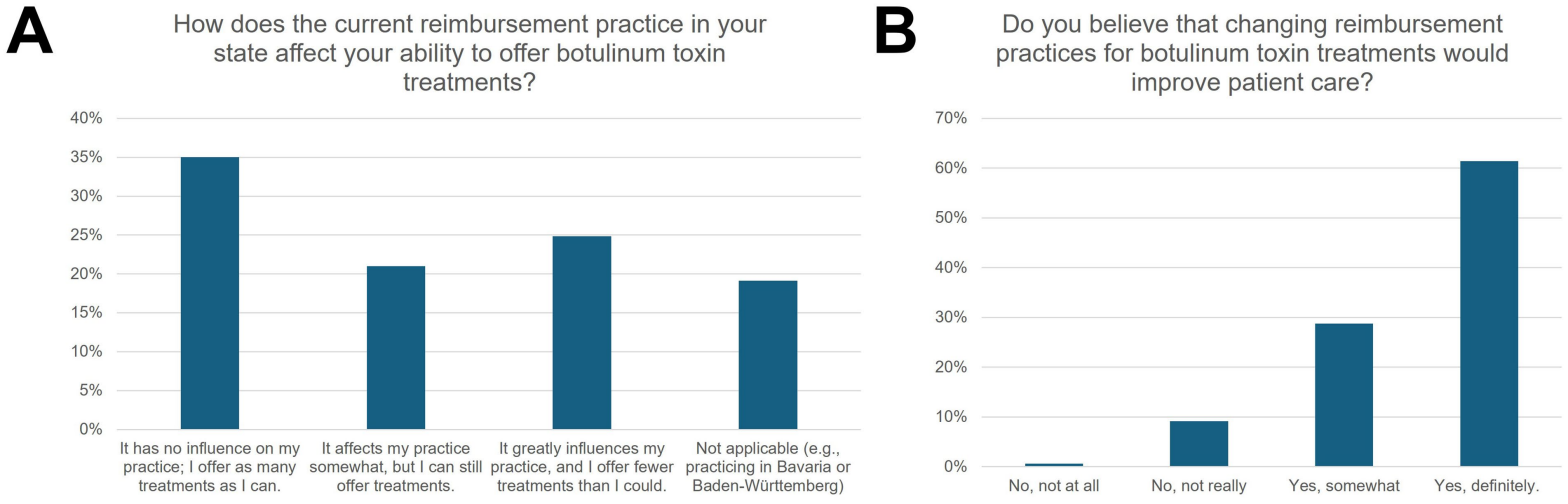
Influence of reimbursement of neurologic botulinum toxin therapy on clinical practice. **(A)** The percentage distribution of answers to the question, how the current reimbursement practice in their state affects the ability to offer botulinum toxin treatments, grouped into no influence, somewhat, and greatly influenced. Respondents also had the option to choose “not applicable.” **(B)** Respondents were also asked if they believe that changing reimbursement practice would improve patient care. Answers were grouped into “No, not at all,” “No, not really,” “Yes, somewhat,” and “Yes, definitely”.

According to 24.8% of the participants, the current reimbursement practice “strongly” influenced their provision of BoNT-A treatments and led to a reduction in the possible scope of treatment (Figure 3). 21.0% saw a partial influence. 61.4% stated that a change in reimbursement modalities would significantly improve care, while another 28.8% expected at least a somewhat better provision of care.

### Patient and disease-specific data

#### Indications and preparations

**Figure 4:**
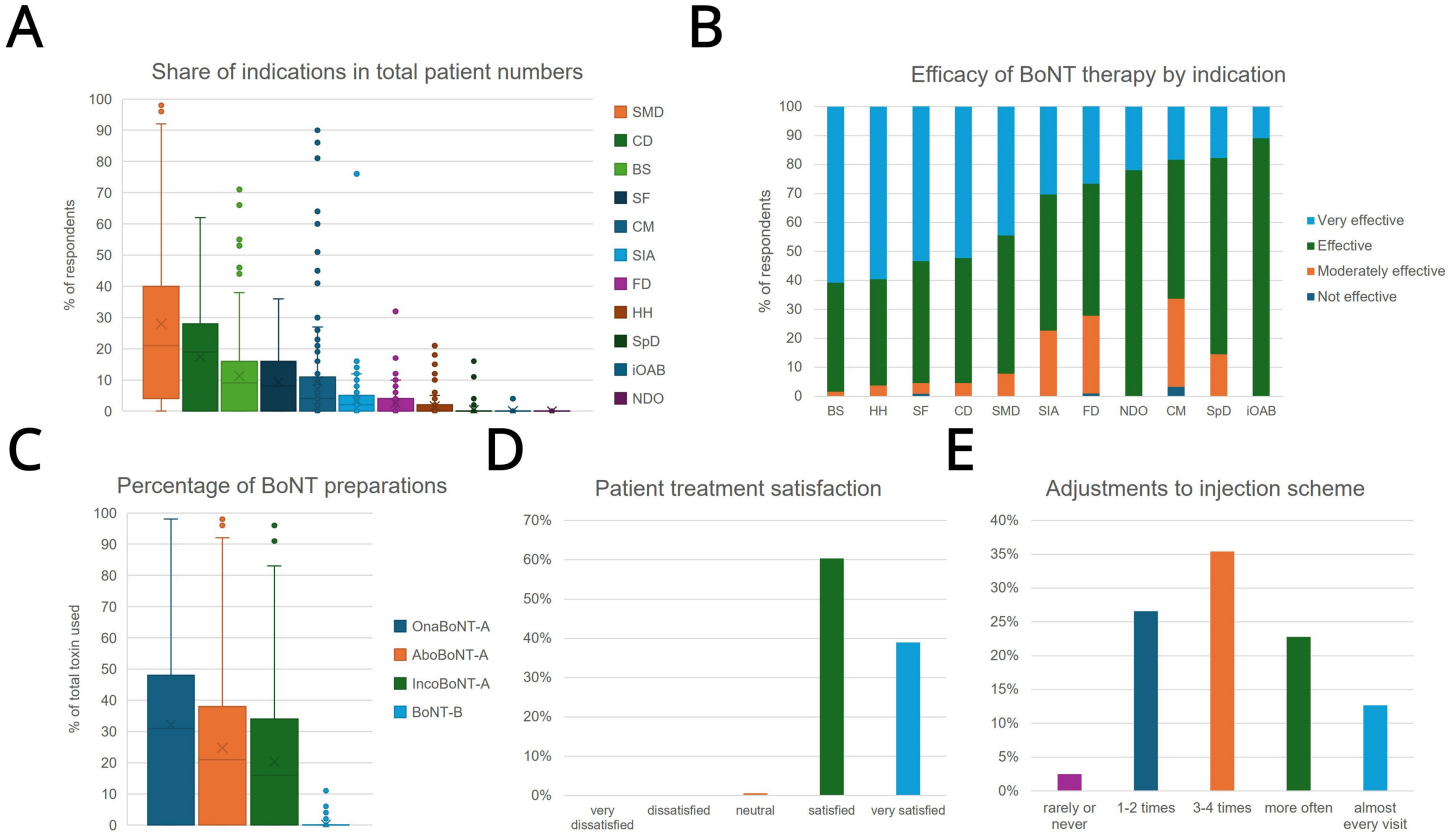
Overview of indications, preparations, and satisfaction with botulinum toxin therapy. **(A)** The percentage distribution of different indications in the total clientele of respondents. **(B)** The perceived effectiveness of botulinum toxin by the practitioner for various indications in four categories (“very effective,” “effective,” “moderately effective,” “not effective”). **(C)** Illustrates the ratio in which the four BoNT preparations used (OnaBoNT-A, AboBoNT-A, IncoBoNT-A, and BoNT-B) are used by the respondents. **(D)** Indicates the proportion of respondents who indicated a respective level based on the practitioners’ assessment of their patients’ satisfaction (“very dissatisfied” to “very satisfied”). **(E)** How often the practitioners change the dosage or injection sites during the course of treatment (“rarely or never,” “1–2 times,” “3–4 times,” “more often,” “almost every visit”). Boxplots visualize median (line), mean (x), and interquartile range. CD, cervical dystonia; BS, blepharospasm; SF, spasmus (hemi-) facialis; CM, chronic migraine; SIA, sialorrhea; FD, task-related focal dystonia; HH, hyperhidrosis; SpD, spasmodic dysphonia; iOAB, idiopathic overactive bladder; NDO, neurogenic detrusor hyperactivity in neurogenic bladder.

The most common indication was spasticity (39.0% of the total patient population), followed by cervical dystonia (24.8%), blepharospasm (16.3%), and CM (15.8%) (Figure 4). Less frequently mentioned were hemifacial spasm (13.6%), sialorrhea (6.8%), and focal dystonias (e.g., task-specific writer’s cramp; 5.3%). Among the preparations used, Onabotulinumtoxin A (OnaBoNTA; 48.99% share), Abobotulinumtoxin A (AboBoNTA; 36.78%), and Incobotulinumtoxin A (IncoBoNTA; 33.19%) dominated, while Botulinumtoxin B (BoNTB) accounted for only 2.94%.

#### Side effects, latency, and duration of effect

Physicians were also asked about the adverse events and treatment effects reported by their patients: The most common side effects reported were muscle weakness in the injected muscle (49.1%) and pain at the injection site (33.3%), while 31.5% observed weakness in adjacent muscles (“spillover effect”). Treatment effect initiared in less than a week for 41.1% of patients, and after one to 2 weeks for 52.1%. 47.2% reported a duration of effect of less than 3 months, while the slight majority (51.5%) reported three to 6 months.

#### Effectiveness in specific patient groups and diseases

More than 80% of respondents rated the therapy as “very effective” or “effective” for most indications. Particularly high effectiveness rates were reported for spastic movement disorders (SMD; 44.49% “very effective,” 47.71% “effective”) and cervical dystonia (CD; 52.29% “very effective,” 43.14% “effective”). The ratings for blepharospasm (BS; 60.82% “very effective,” 37.66% “effective”) and (hemi-) facial spasm (SF; 53.49% “very effective,” 42.04% “effective”) were at a similar level. The information on sialorrhea (SIA; 6.8%), focal dystonias (FD; 5.3%), idiopathic overactive bladder (iOAB), and neurogenic detrusor overactivity (NDO) also indicated a predominantly (very) effective outcome. In contrast, assessments for CM were more differentiated: 18.36% rated the therapy as “very effective,” 48.01% as “effective,” and 30.4% as “moderately effective.” Only 3.23% classified it as “not effective.” Regarding age- and gender-specific differences in therapeutic response, 73.5% were neutral, while 9.9% considered younger patients (<50 years) to be particularly responsive.

#### Treatment adjustment and patient satisfaction

For 26.6% of respondents, the injection dose or sites were adjusted once or twice, 35.4% reported three to four modifications, and 12.7% stated they changed the therapeutic regimen in almost every visit. Overall, 99.4% rated patient satisfaction as “very satisfied” (39.0%) or “satisfied” (60.4%).

## Discussion

Our study adds to the knowledge about the care situation of neurological patients with botulinum toxin in Germany and shows important demographic developments of the practitioners, clinical characteristics of the patients and obstacles in the care of patients despite spatial accessibility.

Even in a well-resourced system with broad geographic access to specialized care, as demonstrated by the accessibility analysis of Neurological Botulinum Toxin Centers in Germany, significant treatment gaps persist from the perspective of treating physicians. Our findings, derived from a German case study, indicate that while BoNT-A is considered a highly effective therapy by expert users, its application is hindered by structural and economic barriers. Even though the generalizability is unclear, we hypothesize, that these challenges are likely not unique to Germany and could offer lessons for an international audience (8).

Our findings align with the situation already described in other studies. For instance, Raffauf and Funke pointed out that an adequate reimbursement practice and a comprehensive network of BoNT-A practitioners are insufficiently established in Germany, with sometimes large regional differences (9). As a consequence, patients who could benefit from BoNT-A therapy according to guidelines (10) are often treated with delay or not at all.

### Discrepancy between guidelines, reality of care, and accessibility analysis

The importance of early and focal therapy of SMD with BoNT-A has been repeatedly emphasized in the current literature (11). Recent analyses of statutory health insurance billing data showed that only about 10% of patients with SMD for whom BoNT-A treatment would be indicated according to guidelines received this first-line therapy (12) and only 1% of patients with PSS received therapy with BoNT-A (13). Barriers to guideline-adherent BoNT-A use in post-stroke spasticity (PSS) include budget constraints, inadequate remuneration, limited practitioner availability, and insufficient guideline awareness (13). Our survey results reflect this, as many participants cited an inadequate reimbursement structure and organizational barriers (e.g., lack of billing codes outside of Bavaria or Baden-Württemberg) as key reasons for the undersupply. At the same time, the accessibility analysis, which was conducted based on the 2022 German Hospital Directory, suggests extensive geographical coverage. According to our calculations, over 99% of the population is in reach of least one specialized center within a 90-min drive. This indicates that the discrepancy is not primarily due to a lack of facilities or excessive travel times. Instead, possible causes arise from structural deficits with insufficient cross-sectoral cooperation and billing obstacles. A lack of awareness of the BoNT-A therapy option or missing referral pathways could also explain the difference between theoretical accessibility and the practical use of BoNT-A services.

Potential implication for other healthcare systems is that establishing specialist centers may be insufficient; the administrative and economic frameworks should support their use. The lack of uniform, nationwide billing codes, with reimbursement varying by region, displays a key barrier identified in Germany and highlights how regional policy disparities can impede patient access even when physical infrastructure is in place.

### Therapeutic efficacy, clinical practice, and patient-reported outcomes

Despite structural obstacles, most of our respondents reported good to very good results with BoNT-A injections across a broad spectrum of indications. The high effectiveness rates reported in our survey for spasticity, cervical dystonia, and blepharospasm, as well as sialorrhea are corroborated by a high level of evidence in scientific literature, including practice guidelines and multiple Cochrane systematic reviews (2, 14–19). Similarly, the effectiveness ratings for chronic migraine - with a spectrum of “very effective” to “moderately effective” correspond to the clinical reality established in trials, which show a significant benefit compared to placebo (20, 21).

Furthermore, our data on treatment adjustments underscore that BoNT-A therapy is a highly individualized process. The finding that nearly half of the physicians surveyed adjust a patient’s regimen three or more times aligns with expert consensus that continuous optimization of dose and injection sites is a hallmark of high-quality care, not an indication of treatment failure in managing cervical dystonia (22) This iterative approach is essential for balancing maximal efficacy with minimal adverse effects.

The overall high satisfaction in our survey confirms the therapeutic value, especially for spasticity, cervical dystonia, and blepharospasm. However, it is important to note that these figures represent the physicians’ assessment of their patients’ satisfaction rather than direct patient-reported outcomes. While this high rating aligns with existing literature (17, 23–25), it may lack the nuance of the patient experience, such as the impact of symptom re-emergence between cycles. Furthermore, clinician-perceived satisfaction does not always correlate with long-term treatment adherence, which can be influenced by cumulative costs, the burden of travel, or the psychological impact of the “waning effect” observed in nearly half of the patients in our data. Future research should incorporate direct patient perspectives to bridge this interpretive gap.

Furthermore, patient satisfaction is not static. It is highest at the time of the treatment’s peak effect and declines toward the end of the injection cycle as symptoms potentially re-emerge (26). This “waning effect” is validated by our own data on treatment duration, which shows that nearly half of patients experience therapeutic effects for less than the standard 12-week interval. This adds further expert opinion for the clinical rationale of more flexible and individualized treatment schedules to maintain patient satisfaction and quality of life.

Most of our respondents saw no age- or gender-specific differences, which speaks for the broad applicability of BoNT-A; the view of some of our respondents that younger patients respond better has not been substantiated by robust evidence, however there have been studies showing distinct dosing and response patterns for female and male patients (27–29).

### Need for further training, junior staff problems, and cross-sectoral networking

A special aspect of our data is the long-standing professional experience of many BoNT-A practitioners. In our sample, about two-thirds had at least 10 years of experience with BoNT-A and often belonged to the age group over 50 years. This demographic structure could potentially lead to a relevant problem with the recruitment of junior staff, as the expertise for BoNT-A therapies has so far been concentrated in the hands of an experienced group of colleagues. Securing the quality of care therefore requires targeted further training measures, especially familiarizing young physicians with injection techniques, dosage principles, and indications. This is consistent with the model training regulations for neurology in Germany (2018), which explicitly require the acquisition of competence in “Botulinum toxin therapy for the treatment of dystonias and spasticity” (30).

### The importance of cross-sectoral collaboration

Another aspect of the undersupply concerns the lack of awareness of BoNT-A indications among non-neurological disciplines, as well as shortages of specially trained personnel. This aligns with findings from the literature, according to which the successful treatment of spasticity and other BoNT-A-relevant indications requires a clear indication, the early establishment of a multiprofessional treatment concept, and the involvement of specialized centers (1, 2). Furthermore, the lack of systematic education and discharge management (for example, for stroke patients in nursing homes) is described as a barrier. It is precisely here that closer communication and cooperation between office-based physicians, inpatient facilities, and specialized centers could reduce the gap between theoretical accessibility and the practical use of BoNT-A services (3).

### Perspective: improvement of reimbursement and structured follow-up

The results of our survey suggest that an improved reimbursement structure and clearly defined billing pathways could lead to an increase in BoNT-A therapy (31, 32). Furthermore, the results suggest that regular training offers could make more physicians willing to perform or expand the therapy. Finally, a cross-sectoral network between specialists, inpatient facilities, and office-based colleagues would be desirable to consistently implement the effectiveness and cost-effectiveness of BoNT-A, confirmed in numerous studies, in Germany as well (33). The high coverage shown in our accessibility analysis could indicate that a suitable infrastructure already exists, which, however, may not be fully utilized.

### Limitations

The validity of the study is limited by its focus on AK-BoNT members, as a selection of experienced users and thus a possible overestimation of the care situation cannot be ruled out. Furthermore, while the survey response rate of 30.2% (191 out of 632) is consistent with similar clinician surveys, it introduces the potential for non-response bias. Clinicians who are more actively engaged with BoNT-A therapy or those experiencing fewer administrative hurdles may have been more motivated to participate. Consequently, the results may not fully generalize to the broader medical community or to practitioners with less specialized experience who might face even greater barriers to therapy utilization.

## Conclusion

Using Germany as a case study, this study suggests that closing the treatment gap for a proven therapy like BoNT-A may require a systemic approach that moves beyond ensuring geographic access. The primary barriers identified in our study with insufficient reimbursement policies, an aging specialist workforce, and poor cross-sectoral integration may be challenges faced by many healthcare systems worldwide.

Based on our findings, we propose the following general principles for improving patient access to specialized neurological care internationally:

- *Standardize Reimbursement:* The development of adequate, and uniform reimbursement structures to remove economic barriers for both patients and providers.
- *Invest in Education and Training:* Health systems should address the challenge of an aging specialist workforce by integrating specialized training into medical education and creating continuous professional development opportunities to ensure a sustainable supply of skilled clinicians.
- *Foster Integrated Care Networks*: Improving communication and creating seamless referral pathways between inpatient and outpatient care facilities is essential to ensure patients are identified and receive continuous treatment.

## Data Availability

The raw data supporting the conclusions of this article will be made available by the authors, without undue reservation.

## Footnotes

1
https://www.deutsches-krankenhaus-verzeichnis.de/app/suche
