# Supplementary material for: Mind the gap: a German case study on the discrepancy between geographic accessibility and real-world utilization of botulinum toxin therapy
Source: Front Neurol. 2026 Feb 18;17:1715279. doi: 10.3389/fneur.2026.1715279 (PMC12956732; doi:10.3389/fneur.2026.1715279)
Supplement: Supplementary file 1 [file Data_Sheet_1.pdf]

Bild

SD01

Umfrage Arbeitskreis Botulinumtoxin

Sehr geehrte Mitglieder des Arbeitskreises Botulinumtoxin,

wir möchten Sie bitten, an einer Umfrage teilzunehmen, die uns dabei helfen soll, ein besseres Verständnis für die Anwendung von Botulinumtoxin in neurologischen Indikationen zu erlangen. Ihre Antworten werden uns wertvolle Einblicke in die aktuellen Praktiken und Trends in diesem Bereich geben und uns dabei unterstützen, unsere Arbeit und Forschung zu verbessern.

Es handelt sich um einen Fragebogen, für dessen Beantwortung Sie nur wenige Minuten Zeit benötigen. Ihre Antworten werden selbstverständlich vertraulich behandelt und nur anonymisiert für die Zwecke dieser Studie verwendet. Wir gehen davon aus, dass Sie, wenn Sie den Fragebogen online beantworten, implizit in die Auswertung dieses Fragebogens einwilligen. Wir planen, die Ergebnisse der Befragung anonymisiert zu publizieren.

SD02

1. Postleitzahl

SD03

2. Fachrichtung

[Bitte auswählen]

SD04

3. Geschlecht

[Bitte auswählen]

SD05

4. Alter

[Bitte auswählen]

Bild

SD06

5. Wie viele Patienten behandeln Sie durchschnittlich pro Jahr mit Botulinumtoxin?

[Bitte auswählen]

SD07

6. Wie viele Jahre behandeln Sie bereits mit Botulinumtoxin A?

[Bitte auswählen]

SD08

7. Welche neurologischen Indikationen behandeln Sie am häufigsten mit Botulinumtoxin? Bitte geben Sie eine ungefähre Prozentzahl für jede Indikation an.

Chronische Migräne in %

0 100

Spastik in %

0 100

Zervikale Dystonie in %

0 100

Spasmodische Dysphonie in %

0 100

Aufgabenspezifische fokale Dystonie in %

0 100

Blepharospasmus in %

0 100

Spasmus hemifacialis in %

0 100

Sialorrhoe in %

0 100

Idiopathische überaktive Blase in %

0 100

Neurogene Detrusorhyperaktivität bei neurogener Blase in %

0 100

Hyperhidrosis axillaris in %

0 100

8. Wie hat sich die Anzahl der Patienten, die Sie mit Botulinumtoxin behandeln, in den letzten fünf Jahren verändert? SD09

- ◯ Erhöht
- ◯ Verringert
- ◯ Unverändert

9. Welche Formulierungen von Botulinumtoxin verwenden Sie am häufigsten in Ihrer Praxis? SD10

Bitte geben Sie den Anteil in % an.

	0 100
OnaBONT-A (BOTOX)	
AboBONT-A (DYSPORT)	
IncoBONT-A (XEOMIN)	
BONT-B (NEUROBLOC)	

Bild

SD11

10. Wie beurteilen Sie (im Vergleich zu anderen Indikationen) die Wirksamkeit von Botulinumtoxin.

	Sehr effektiv	Effektiv	Mäßig effektiv	Nicht effektiv	Ich behandle diese Erkrankung nicht.
Chronische Migräne	○	○	○	○	○
Spastik	○	○	○	○	○
Zervikale Dystonie	○	○	○	○	○
Spasmodische Dysphonie	○	○	○	○	○
Aufgabenspezifische fokale Dystonie	○	○	○	○	○
Blepharospasmus	○	○	○	○	○
Spasmus hemifacialis	○	○	○	○	○
Sialorrhoe	○	○	○	○	○
Idiopathische überaktive Blase	○	○	○	○	○
Neurogene Detrusorhyperaktivität bei neurogener Blase	○	○	○	○	○
Hyperhidrosis axillaris	○	○	○	○	○

SD12

11. Welche Nebenwirkungen beobachten Sie am häufigsten bei der Behandlung mit Botulinumtoxin? (Mehrfachauswahl möglich)

☐ Störende Muskelschwäche des injizierten Muskels

☐ Störende Muskelschwäche von direkt den injizierten Muskeln benachbarten Muskeln

☐ Muskelschwäche an von den injizierten Muskeln entfernen Orten (distant spread)

☐ Schmerzen an der Injektionsstelle

☐ Kopfschmerzen

☐ Grippeähnliche Symptome

☐ Andere

SD13

12. Welche Ressourcen oder Schulungen würden Sie hilfreich finden, um Ihre Praxis in Bezug auf die Anwendung von Botulinumtoxin zu verbessern? (Mehrfachauswahl möglich)

☐ Hospitation in einem Zentrum

☐ Vor Ort Hands-on Workshops

☐ Webinare

☐ Fachliteratur

☐ Andere

SD14

13. Wie lange dauert es in der Regel, bis Ihre Patienten eine Wirkung nach der Botulinumtoxin-Behandlung verspüren?

○ Weniger als eine Woche

○ 1-2 Wochen

○ 2-4 Wochen

○ Mehr als 4 Wochen

SD15

14. Wie lange hält die Wirkung der Botulinumtoxin-Behandlung bei Ihren Patienten in der Regel an?

- ◯ Weniger als 3 Monate
- ◯ 3-6 Monate
- ◯ 6-9 Monate
- ◯ Mehr als 9 Monate

Bild

SD16

15. Haben Sie festgestellt, dass bestimmte Patientengruppen (z.B. nach Alter, Geschlecht, allgemeinem Gesundheitszustand) besser auf die Botulinumtoxin-Behandlung ansprechen? (Mehrfachauswahl möglich)

- ☐ Ja, Jüngere (< 50 Jahre)
- ☐ Ja, Ältere (≥ 50 Jahre)
- ☐ Ja, Männer
- ☐ Ja, Frauen
- ☐ Ja, nach allgemeinem Gesundheitszustand
- ☐ Nein, keine bemerkenswerten Unterschiede

SD17

16. Haben Sie festgestellt, dass die Botulinumtoxin-Behandlung bei bestimmten neurologischen Indikationen effektiver ist?

- ○ Ja, bei Spastik
- ○ Ja, bei Dystonie
- ○ Ja, bei chronischer Migräne
- ○ Ja, bei Hyperhidrose
- ○ Nein, keine bemerkenswerten Unterschiede

SD18

17. Bitte bringen Sie die einzelnen Indikationen in eine Reihenfolge von am wenigsten effektiv (1) bis hin zu am effektivsten (7).

Spastik	Dystonie	1
Chronische Migräne	Hyperhidrose	2
	Sialorrhoe	3
Blepharospasmus	Spasmus (hemi)facialis	4
		5
		6
		7

SD19

18. Wie oft passen Sie die Botulinumtoxin-Behandlung in der Regel an (z.B. Dosierung, Injektionsstellen), um eine optimale Wirkung zu erzielen?

- ○ Selten oder nie
- ○ 1-2 mal
- ○ 3-4 mal
- ○ öfter
- ○ Fast bei jeder Visite

SD20

19. Wie beurteilen Sie die Zufriedenheit Ihrer Patienten mit der Botulinumtoxin-Behandlung?

- ○ Sehr zufrieden
- ○ Zufrieden
- ○ Neutral
- ○ Unzufrieden
- ○ Sehr unzufrieden

Bild

SD21

20. Wie beeinflusst die aktuelle Vergütungspraxis in Ihrem Bundesland Ihre Fähigkeit, Botulinumtoxin-Behandlungen anzubieten?

- ◯ Sie beeinflusst meine Praxis stark und ich biete weniger Behandlungen an, als ich könnte
- ◯ Sie beeinflusst meine Praxis etwas, aber ich kann immer noch Behandlungen anbieten
- ◯ Sie hat keinen Einfluss auf meine Praxis, ich biete so viele Behandlungen an, wie ich kann
- ◯ Nicht zutreffend (z.B. in Bayern oder Baden-Württemberg tätig)

SD22

21. Glauben Sie, dass eine Änderung der Vergütungspraktiken für Botulinumtoxin-Behandlungen die Patientenversorgung verbessern würde?

- ◯ Ja, deutlich
- ◯ Ja, etwas
- ◯ Nein, nicht wirklich
- ◯ Nein, überhaupt nicht

SD23

22. Haben Sie Vorschläge oder Anmerkungen, wie die Unterstützung für Praktiker wie Sie verbessert werden könnte?

Bild

SD24

Vielen Dank für Ihre Zeit und Ihre wertvollen Beiträge. Ihre Meinung ist uns sehr wichtig und wird dazu beitragen, die Qualität der Patientenversorgung zu verbessern.

Mit freundlichen Grüßen

Prof. Dr. Philipp Albrecht (Neurologie Kliniken Maria Hilf Mönchengladbach und Heinrich-Heine-Universität)
PD Dr. John-Ih Lee (Neurologie Unversitätsklinikum Düsseldorf und Heinrich-Heine-Universität)
Dr. Tristan Kölsche (Neurologie Unversitätsklinikum Düsseldorf und Heinrich-Heine-Universität)
Valeria Koska (Neurologie Kliniken Maria Hilf Mönchengladbach)

Klinik für Neurologie
Universitätsklinikum Düsseldorf (UKD) - Anstalt des öffentlichen Rechts
Moorenstraße 5
40225 Düsseldorf
Tel.: +49 211 81-00
Fax: +49 211 81-04855
Internet: www.uniklinik-duesseldorf.de
E-Mail: uniklinik.redaktion@uniklinik-duesseldorf.de

Klinik für Neurologie
Kliniken Maria Hilf GmbH
Viersener Straße 450
41063 Mönchengladbach
Tel.: (02161) 892-0
Fax: (02161) 892-2080
info@mariahilf.de
Kliniken Maria Hilf GmbH Mönchengladbach

———

Universitätsklinikum Düsseldorf (UKD) - Anstalt des öffentlichen Rechts
Aufsichtsratsvorsitzender: Prof. Dr. Dr. h.c. Peter Dominiak
Vorstand: Prof. Dr. Dr. Frank Schneider (Ärztlicher Direktor, Vorstandsvorsitzender) | Ekkehard Zimmer (Kaufmännischer Direktor, stellv. Vorstandsvorsitzender) | Prof. Dr. Benedikt Pannen (stellv. Ärztlicher Direktor) | Torsten Rantzsch (Pflegedirektor) |
Prof. Dr. Nikolaj Klöcker (Dekan der Medizinischen Fakultät)

Steuernummer: 106-5773-0045
Bankverbindung: Stadtsparkasse Düsseldorf | BLZ: 300 501 10 | Kto.-Nr.: 10 001 550 | IK: 26051 00 18 |
SWIFT-BIC: DUSSDEDDXXX | IBAN: DE 94 3005 0110 0010 001550

Disclaimer: Die in dieser Umfrage und den dazugehörigen Anhängen enthaltenen Informationen sind nur für den Adressaten bestimmt und können vertrauliche und/oder rechtlich geschützte Informationen enthalten. Sollten Sie die Nachricht

irrtümlich erhalten haben, löschen Sie die Nachricht bitte und benachrichtigen Sie den Absender, ohne die Nachricht zu kopieren oder zu verteilen oder ihren Inhalt an andere Personen weiterzuleiten.

Letzte Seite

Ihre Antworten wurden gespeichert, Sie können das Browser-Fenster nun schließen.

Mit freundlichen Grüßen

Prof. Dr. Philipp Albrecht (Neurologie Kliniken Maria Hilf Mönchengladbach und Heinrich-Heine-Universität)

PD Dr. John-Ih Lee (Neurologie Unversitätsklinikum Düsseldorf und Heinrich-Heine-Universität)

Dr. Tristan Kölsche (Neurologie Unversitätsklinikum Düsseldorf und Heinrich-Heine-Universität)

Valeria Koska (Neurologie Kliniken Maria Hilf Mönchengladbach)

{EM_COPYRIGHT} Arbeitskreis Botulinumtoxin 2023 im Netzwerk der Deutschen Gesellschaft für Neurologie (DGN)
